# Role of Implantable Cardioverter Defibrillators in the Treatment of Hypertrophic Cardiomyopathy

**Published:** 2005-04-01

**Authors:** Joydeep Ghosh, Johnson Francis, Barry J Maron

**Affiliations:** * Mount Sinai School of Medicine, Maimonides Medical Center, Brooklyn, New York, USA; † Medical College Calicut, Kerala, India; ‡ The Hypertrophic Cardiomyopathy Center, Minneapolis Heart Institute Foundation, Minneapolis, USA

**Keywords:** Hypertrophic cardiomyopathy, implantable defibrillator, sudden cardiac death

## Abstract

Hypertrophic cardiomyopathy (HCM) is an important cardiovascular disease with sudden cardiac death as the most devastating presentation. Implantable cardioverter defibrillators (ICD) are the optimal therapy for prevention of sudden death from ventricular tachycardia or fibrillation of any cause. While there is no controversy with implanting ICDs in patients who have already survived a cardiac arrest, identifying high-risk patients for primary prevention in this disease remains a challenge. Implanting ICDs in patients with HCM is an important clinical consideration since many individuals could achieve normal or near-normal lifespans with this protection.

Hypertrophic cardiomyopathy (HCM) is the most common genetically inherited cardiac disorder with an estimated prevalence of 1 in 500 in the general population [1]. The disease is characterised by marked heterogeneity with respect to clinical manifestations, natural history and prognosis. Sudden cardiac death (SCD) is the most the devastating consequence of the disease and HCM is now recognized as most frequent cause of sudden cardiac death in pre-adolescent and adolescent children, as well as young athletes. This is not to suggest, however that patients who have reached midlife or even beyond are immune from SCD; in fact, no age is considered safe from this point-of-view [2,3].

The causes of sudden death in HCM remain controversial. While earlier theories outflow-tract obstruction, addressedthe current consensus favors primary ventricular arrhythmias, while recognising that the pathogenesis of such arrhythmias is multifactorial and involve complex interactions between a combination of factors. Given the high liklihood of prevention of sudden death from ventricular tachycardia or ventricular fibrillation by implantable cardioverter-defibrillators (ICD) in multiple primary and secondary prevention trials of ischemic and non-ischemic dilated cardiomyopathy, it is a reasonable expectation that such results can be replicated in patients with HCM. The difference between HCM and coronary artery disease is that risk-stratification in HCM with such variable natural history is exceedingly difficult and there exists no definitive consensus on precisely which patients should receive an ICD for primary prevention. On the other hand the ICD is strongly indicated for any patient, including those with HCM, who has survived a documented cardiac arrest i.e., secondary prevention [4, 5].

It is important to remember that unlike other patients with ischemic or non-ischemic cardiomyopathy and ICD implants who have a limited life-span owing to advanced congestive heart failure and age, high risk HCM patients are usually younger and asymptomatic. Consequently, the potential of the ICD to prolong life is much greater in the HCM population, and therefore it is vitally important that patients be identified correctly for this therapy. In order to create a high risk profile, all HCM patients should undergo an initial comprehensive ambulatory risk stratification assessment, including detailed personal and family history and physical examination, 12-lead ECG, echocardiogram, ambulatory Holter monitoring and exercise testing. The currently recognised major risk factors for SCD in HCM include: unexplained syncope, (particularly when exertional or recurrent), family history of HCM-related sudden death, identification of high-risk mutant genes, frequent, multiple or prolonged  episodes of non-sustained ventricular tachycardia on Holter monitoring, abnormal blood pressure response to exercise and extreme degrees of left ventricular hypertrophy (maximum left ventricular wall thickness of 30 mm or more), particularly in adolescents and young adults [6]. Invasive electrophysiology study as a means of risk stratification has been largely abandoned in HCM patients, primarily because of the unknown clinical significance of commonly induced ventricular tachyarrhythmias in the laboratory.

Controversy exists as to how many major risk factors are required in a particular patient to be considered deserving of an ICD, and some of this is uncertainty is due to the fact that the magnitude of risk conferred by each risk marker may not be the same. While the presence of multiple risk factors may convey an increased risk, a single risk factor may be adequate in a given patient to justify an ICD implant. ICD trials in HCM have documented their efficacy for sensing and terminating arrhythmias. A recent study by Maron, et al. showed appropriate discharges triggered by VT in 23% of patients when followed over 3 years, with an average discharge rate of 5% per year for primary prevention and 11% per year for secondary prevention. About 60% of the patients with appropriate interventions received multiple discharges [7].

There are certain technical considerations for the electrophysiologist implanting ICD's in this population that deserve mention. The first relates to whether a single- or dual-chamber system should be employed. Dual chamber devices have the benefit of AV synchrony with pacing, although for the most part pacing as a treatment modality in HCM to reduce symptoms and outflow gradient was largely abandoned after the M-PATHY trial [8]. More importantly, dual chamber devices provide superior SVT discrimination. The latter is particularly important in this population which has  a high incidence of atrial fibrillation, though newer technology (for morphology, stability and onset discrimination) may provide relatively good discrimination even with single lead systems. The single-chamber “shock box” device is probably a more appropriate option for young patients who do not appear susceptible to atrial fibrillation and in whom survival depends largely on effective ventricular arrhythmia termination. A single chamber ICD is also indicated in all patients with chronic atrial fibrillation, while patients with paroxysmal atrial fibrillation would probably benefit more from dual chamber devices with mode-switch capability. It should be emphasized that atrial fibrillation may be poorly tolerated in HCM patients who already have severe diastolic dysfunction since LV filling is critically dependent on effective atrial systole. A dual chamber device with rate-drop response is probably a better option for a HCM patient with recurrent episodes of neurocardiogenic syncope, although trials of pacing for neurocardiogenic syncope have so far provided ambiguous results. An excellent update on selection of ICDs for each patient has been published recently [9].

Lead fractures and device recalls are particularly relevant events that affect young HCM patients who will likely spend their whole lives with the ICD system. Unfortunately, these are impossible to predict in advance, and lead removal, in particular for older leads, is fraught with difficulties [10]. Whenever possible, adding a new lead should be preferred to extracting an old one. Though active fixation leads that are less than 6-months old are usually removed without difficulty, passive leads require laser excision and should only be performed in a center with considerable experience with such procedures.

The number of HCM patients who meet criteria for an ICD implant is increasing. It behoves the cardiovascular community to offer these patients all major advances in treatment so that they can continue to lead productive lives, given their preserved systolic function and often minimum symptoms. Many of the current uncertainties about patient selection will be resolved soon with further clinical trials, and in the future more HCM patients will live longer lives with the aid of more technologically advanced ICD systems.
